# A systematic review of the childbearing needs of single-child couples

**DOI:** 10.1186/s12905-024-02928-0

**Published:** 2024-02-01

**Authors:** Fatemeh Seraj Shirvan, Maryam Moradi, Robab Latifnejad Ruodsari

**Affiliations:** 1 Student Research Committee, Mashhad University of Medical Sciences, Mashhad, Iran; 2 SPHERE-NHMRC Centre of Research Excellence, Department of General Practice, Faculty of Medicine, Nursing and Health Sciences, Monash University, Melbourne, Victoria Australia; 3 Nursing and Midwifery Care Research Center, Mashhad University of Medical Sciences, Mashhad, Iran; 4 Department of Midwifery, School of Nursing and Midwifery, Mashhad University of Medical Sciences, Mashhad, Iran

**Keywords:** Only child, Reproductive behavior, Needs assessment

## Abstract

**Background:**

Nowadays, the challenge of having single child is spreading in many countries. Only- child family is prevalent in 26% of families in Canada, 21% in the United States, 47.5% in Europe and 20% in Iran, which can lead to fertility below replacement level. Therefore, the current review was conducted to identify the needs of single-child couples.

**Methods:**

The PRISMA checklist was used to prepare this systematic review report. English and Persian articles published between 2000 and April 2023 were searched in the English databases of ISI, PubMed, Cochrane library and Google Scholar search engine as well as Persian databases of SID and Magiran using keywords of only child, needs assessment and Reproductive behavior. All cross-sectional and correlational studies that addressed the needs of single-child couples were included in the study. The quality assessment of the articles was done by the STROBE checklist. Data extraction was done by two independent researchers using a self-structured checklist. To analyze the data, following tabulating the extracted data, the process of qualitative synthesis was done for systematic review taking into account ethical considerations.

**Results:**

Out of 1,581 articles found, 17 articles were included in the systematic review. The needs of single-child couples were divided into four general areas included 1) Financial needs, 2) Cultural needs, 3) Educational needs, and 4) Supportive needs. The support needs included two kinds of social and family support.

**Conclusion:**

Solving financial needs, creating a culture of positive values of childbearing and men's participation in household affairs, considering women's preferences in order to increase education and employment, childbearing training and counseling and creating social and family support in line with work and family harmony and quality care of children, as the most important needs of single-child couples, should be incorporated in the formulation of childbearing incentive programs.

**Supplementary Information:**

The online version contains supplementary material available at 10.1186/s12905-024-02928-0.

## Background

In recent years, single-child family has been common in large urban communities, and many families have only one child. In fact, today fertility below replacement level has become common and most of the countries in the world including both high and low and middle income countries, experience it. Along with the globalization of fertility patterns and behaviors, Iran has also experienced extensive changes, so that during the last three decades, fertility rate has significantly decreased in Iran [1].

Approximately 122 countries have fertility rates below the replacement rate. The mean rate of single-child in European countries is 47.5%. Portugal ranks first with 57% of single-child families. In the UK, in 2021 single-child families were 42.5%. In 2019, 26% of families in Canada, 21% in the United States, and 20% in Iran were single-child families. In Asia, the mean of Total fertility rate (TFR) is about 3, which significantly decreased from 1970 to 2019. For example, in Singapore in 2020, 24% of couples were single-child [1].

Recent research shows that fertility differences in European countries cannot be fully explained only by differences in postponing pregnancy, but structural and cultural changes which occur with economic development likely affect fertility decisions not only in terms of timing, but also in terms of quantum [2]. For example, Lucy-Greulich and Tionon (2013) stated that increasing fertility rate to replacement levels occur only in highly developed countries where women's employment is associated with economic development and highlights the importance of structural improvements, especially among working mothers, to mix work and family life [3].

Although it is argued that fertility below replacement level may reflect a general preference for low fertility among couples, the latest survey data for European countries suggest that there are barriers preventing parents from achieving optimal fertility, despite the fact that there are consistent preferences for the two-child family model for women and men in all European countries, independent of the national fertility level [4]. Dalbis (2017) stated that childcare services are an important determinant for transition to the birth of a second child [4].

Lupi (2014) in a study in Australia stated that decrease in life satisfaction in new parents in different areas of life causes a significant decrease in childbearing. In fact, having a second child in mothers was positively related to their satisfaction with job prospects and work-family balance, while for fathers, fertility expectations were positively related to their financial status [5]. Single-child families in India do not accept the responsibility of having children due to "wishing for a high quality life for their children", and idealism in raising children is a fundamental factor in the intention to have only one child [6].

In Iran, similar to other low and middle income countries, due to the increase in women's education and women's employment, a second demographic transition has occurred, and single-child is one of the reasons for decrease in fertility. In the study by Mubasheri (2013), three factors "increasing costs and economic pressure", "lack of support and economic facilities from the government" and the wrong attitude "having more children is a sign of poor social culture" are effective in childbearing [7]. Khalajabadi (2015) stated that choosing to have only one child is a solution to balance the personal, marital, family and environmental conditions of the couple and to match the attitudes and feelings and individual skills with the environment without support in the current society [8].

In a review carried out by Hashemzadeh (2021) entitled "Tendency to childbearing and related factors" by Bronfenbrenner's ecological model, it was stated that couples within the systems of microsystem, mesosystem (family and peer network), exosystem (occupational characteristics, urban residence location, housing situation), macrosystem (cultural and social principles with wider effects on couple system) are influenced by these factors [9]. The findings of a review by Razeghi (2020) showed that, two children is the dominant pattern of the desired number of children, which indicates the convergence of fertility ideals in Iran; and if favorable conditions for childbearing are provided, fertility can be maintained at the replacement level [10]. The gap in the performed reviews indicates the lack of attention to specifically identifying the needs of single-child couples. Therefore, it is necessary to conduct a systematic review focused on the needs of single-child couples.

In fact, most of the pronatalist policies aimed to reduce the factors affecting the conflict between work and motherhood, such as maternity leave, child care, training of early childbearing, and pregnancy at a young age, but it seems essential that the research examines the influence of these policies in each country with its special culture [11]. The existing gap in these policies is due to the lack of needs assessment of single-child couples to develop childbearing incentive programs around the world. In other words, designing programs with a guarantee of implementation in the field of childbearing firstly requires understanding the needs of single-child couples.

A much more important point that highlights the necessity and importance of this study is focus on the outstanding research gap about this emerging phenomenon; because identifying the needs of single-child couples is a necessary condition for implementing interventions to adjust the single-child problems and population decline, which still remains as an unsolved problem. Therefore, it was decided to conduct this study to identify the childbearing needs of single-child couples.

## Methods

In this study, the PRISMA Preferred Reporting Items for Systematic Reviews and Meta-Analyses [12] was used to prepare this systematic review report. Considering that only observational studies were reviewed, MOOSE (Meta-analyses Of Observational Studies in Epidemiology) [13] checklist was also used; as it is specific to review of observational studies, so every element, which was likely to be applicable to this study, was covered in more detail.

All English and Persian articles published in peer-reviewed journals which were conducted in a quantitative observational manner and examined the childbearing needs of single-child couples were searched in the period from 2000 to April 2023 in the databases of ISI, PubMed, Cochrane library and Google Scholar search engine as well as Persian language databases of SID and Magiran with the keywords of one child family, single child family, only child family, childbearing motivation, childbearing, fertility, motivation , need, requirement, demand, want, requisite, challenge, obstacle and their synonyms and Persian equivalents. In order to reach the desired number of articles, a manual search was also done in the references of the retrieved articles. An example of the search strategy is given in Table 1.

**Table 1:** Sample of search strategy

**Concept1**		**Concept2**		**Concept3**
one child family OR	AND	childbearing intention OR	AND	Needs assessment OR
single child family OR		reproductive behavior motivation OR		requirement OR
only child family		childbearing preferences		demand OR
				want OR
				requisite

**Fig. 1:**
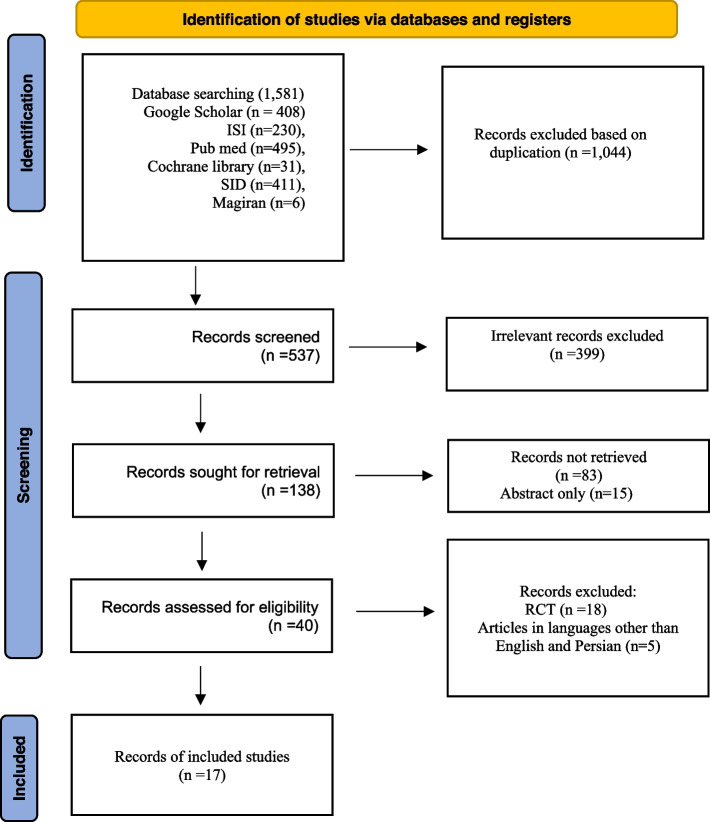
PRISMA 2020 Flow diagram of study selection

Two authors (FSS and MM) independently searched and screened 1,581 studies according to the inclusion criteria. In this way, 1,044 duplicate articles were removed. Then the titles and abstracts of remained articles (537) were reviewed. After excluding of irrelevant and records not retrieved, 40 related full text articles were assessed for eligibility and finally 17 studies were included in the systematic review. Any disagreement between the two authors was discussed by the third author (RLR) and an agreement was achieved. The process of review and selection of articles is given in Fig. 1.

### Inclusion and exclusion criteria

Inclusion criteria were all observational studies including descriptive (survey), cross-sectional, longitudinal and correlational studies in Persian and English. The components of the PECO model were as follows:

- Participants: Single-child couples
- Exposure: Having only one-child
- Control: None
- Outcomes: Needs

Exclusion criteria were articles without free access to the full text, review articles, commentaries, letters to the editor, conference abstracts, as well as guidelines and book chapters related to the single-child families.

### Data extraction

Data extraction was done by two independent researchers (FSS and MM) using a self-structured checklist. This checklist consisted of four parts: general information of the article (name of the authors, year of publication, study setting), participants' characteristics (age, sample size), details of the study method (type of study, number of groups, type of control, data collection tools), and the outcomes (needs, desires) (Table 2). In case of disagreement between the two researchers in data extraction, the senior researcher (RLR) examined the articles and announced the final opinion.

**Table 2:** Descriptive summary of included studies

ID	Study/year	Country	Design of the study	Sample/ Sample size	Age of participants	Perceived need for childbearing	Type Of Need
1	D’Albis 2017 [4]	26 European countries	cross-sectional wave of the EU-SILC( European Union Statistics on Income and Living Conditions (EU-SILC)	Women: 22,143	38-44 y	Need for childcare services Increase in women's wages	Supportive: Women's Socio-Occupational support. Financial
2	Rutigliano R, Lozano M 2022 [14]	Spain	Cross-sectional	Women: 14556 Men: 2619	18-55 y	Grandparent support and informal care	Supportive: Family support
3	Luksik I, Bianchi G. 2016 [15]	Slovakia	Correlational	Women: 1,414	24-36 y	Need to use the TPB (theory of planned behavior) model to create the intention to have a second child	Educational
4	Xu X, Zuo H, Shi Z, et al 2017 [16]	China	Cross sectional	Pregnant women: 2345	>18 y	Teaching the benefits of children or love for children Increase income Solving the problem of unemployment	Educational Financial
5	Jingyue Zhang. 2020 [17]	China	Correlational	Women: 1,834	20-45 y	Direct parenting costs and second child anxiety Job concern Paying attention to the availability of entertainment time and place on the anxiety of having a second child in women	Financial Supportive: Socio-Occupational support
6	Breton D, Prioux F. 2009 [18]	France	Two complementary household surveys	Men and women: 109,602	20-45 y	Balance between work and family for women Culture building for the next generation Participation in religious meetings	Educational Cultural
7	Nagase N, Brinton M. 2017 [19]	Japan	Japanese longitudinal survey	Men and women: 20,486	20-34 y	Increasing the participation of men in housework and the need for changes in the labor law	Supportive Men's Socio-Occupational support
8	Wang q, sun x. 2020 [20]	China	Online survey, Correlational	Men and women: 984	18-40 y	Good economic situation Lowering the age of marriage and first childbearing Reducing the spacing between children "Cultural factors"	Financial Cultural
9	Mansour F 2018 [21]	United States	Correlational	Women: 14,307	21-35 y	The need to solve economic insecurity	Financial
10	Levin V, Besedina E. 2016 [22]	Russia	Correlational	Women: 3,292	15- 44 y	Having a stable job Availability, affordability and quality of formal childcare Teaching ways to empower the combination of work and family Housing provision Improving the quality of marital relationships	Financial Supportive Socio-Occupational support Educational
11	Hwang W, Kim S. 2021 [23]	South Korea	Correlational	Women: 488	18-44 y	Culturalization of men's participation in child care. Creating egalitarian gender attitudes Learning appropriate parenting knowledge for fathers at the birth of their first child.	Educational
12	Liu J, Liu M, Zhang S. 2020 [24]	China	Cross-sectional	Women: 11, 991	18-49 y	Removing economic, parenting (educational and educational) and health barriers to fertility	Financial Educational Supportive: Social health care support
13	Zhu C, Yan L 2022 [25]	China	Cross-sectional	Couples: 1,026	20-45 y	Prevention of late pregnancy, Spacing 3 to 6 years from the first child. Child allowance and Children's educational barriers	Financial Educational
14	Validova A 2018 [26]	Russia	Correlational	Women:35,402	15-49 y	Access to formal childcare options	Supportive Socio-Occupational support
15	Yoon SY. 2017 [27]	Korea	Correlational	Women: 526	<41 years	Supporting husbands, parents or husband's family to do housework and child care	Supportive Family support by husbands
16	Mobasheri M, Alidousti M. 2013 [7]	Iran	Cross-sectional	Women: 180	18-44 y	Reducing costs and economic pressures Government support and facilities for having children Creating the right attitude to childbearing	Financial Educational
17	Basu AM, Desai S. 2016 [6]	India	The India human development survey of 2004–2005 (IHDS). Correlational	women: 33,524	15-49 y	Counseling for self-actualization and personal freedoms of parents	Educational

### Quality assessment

The quality assessment of the articles was done by STROBE checklist (Table 3), which includes 22 items and the main items of cross-sectional and observational studies are assessed by this tool. The overall quality of the articles is determined using this scoring system: when there are no checklist items in the article, a score of zero is assigned to it, and a score of 1 is assigned when every item is present in the article. Obtaining 75% of the total grade is classified as good quality; the grade between 25% and 75% is considered as moderate quality and a grade less than 25% is placed in the poor quality category [28].

**Table 3:** Quality assessment of the included studies in the systematic review using STROBE criteria

Criteria/study	1	2	3	4	5	6	7	8	9	10	11	12	13	14	15	16	17
1a (- indicate the study design	+	+	+	+	+	-	-	+	+	+	+	+	+	+	+	+	+
1b (- An informative and balanced summary	+	-	-	+	-	-	+	-	+	+	+	+	+	+	+	+	+
2) Background/rationale	+	+	+	+	+	+	+	+	+	+	+	+	+	+	+	+	+
3) Objectives	+	+	+	+	+	+	+	+	+	+	+	+	+	+	+	+	+
4) Study design	+	+	+	+	+	+	+	+	+	+	+	+	+	+	+	+	+
5) Setting	+	+	+	+	+	+	+	+	+	+	+	+	+	+	+	+	+
6a) Eligibility criteria, selection of participants, follow-up	+	+	+	+	+	+	+	+	+	+	+	+	+	+	+	+	+
6b) Matching criteria and number of exposed andunexposed	NA^a^	NA	NA	NA	NA	NA	NA	NA	NA	NA	NA	NA	NA	NA	NA	NA	NA
7) Variables	+	+	+	+	+	+	+	+	+	+	+	+	+	+	+	+	+
8) Data sources/measurement	+	+	+	+	+	+	+	+	+	+	+	+	+	+	+	+	+
9) Bias	-	+	+	+	-	+	-	-	+	-	+	+	-	-	-	-	-
10) Study size	+	+	+	+	+	+	+	+	+	+	+	+	+	+	+	+	-
11) Quantitative variables	+	+	+	+	+	+	+	+	+	+	+	+	+	+	+	+	+
12a) All statistical methods	+	+	+	+	+	+	+	+	+	+	+	+	+	+	+	+	+
12b) Subgroups and interactions	+	+	+	+	+	+	+	+	+	+	+	+	+	+	+	+	+
12c) Missing data	NA	NA	NA	NA	NA	NA	NA	NA	NA	NA	NA	NA	NA	NA	NA	NA	NA
12d) loss to follow-up, matching, analytical methods	NA	NA	NA	NA	NA	NA	NA	NA	NA	NA	NA	NA	NA	NA	NA	NA	NA
12e) Sensitivity analyses	NA	NA	NA	NA	NA	NA	NA	NA	NA	NA	NA	NA	NA	NA	NA	NA	NA
13a) Numbers of individuals	+	+	+	+	+	+	+	+	+	+	+	+	+	+	+	+	+
13b) Reasons for non-participation	NA	NA	NA	NA	NA	NA	NA	NA	NA	NA	NA	NA	NA	NA	NA	NA	NA
13c) flow diagram	-	-	-	-	-	-	-	-	-	-	-	-	-	-	-	-	-
14a) Characteristics of study participants	+	+	+	+	+	+	+	+	+	+	+	+	+	+	+	+	+
14b) Participants with missing data	NA	NA	NA	NA	NA	NA	NA	NA	NA	NA	NA	NA	NA	NA	NA	NA	NA
14c) Summaries follow-up time	NA	NA	NA	NA	NA	NA	NA	NA	NA	NA	NA	NA	NA	NA	NA	NA	NA
15) Outcome data	+	+	+	+	+	+	+	+	+	+	+	+	+	+	+	+	+
16a) Unadjusted estimates	-	-	-	-	-	-	-	-	-	-	-	-	-	-	-	-	_
16b) Category boundaries	+	-	-	+	+	+	+	+	+	+	+	+	+	+	+	+	+
16c) Relative risk	+	NA	NA	+	NA	+	NA	NA	NA	NA	NA	+	+	NA	NA	NA	+
17) Other analyses	+	+	+	+	+	+	+	+	+	+	+	+	+	+	+	+	+
18) Key results	+	+	+	+	+	+	+	+	+	+	+	+	+	+	+	+	+
19) Limitations	+	+	-	+	+	-	-	-	-	-	+	+	-	+	+	-	-
20) Interpretation	+	+	+	+	+	+	+	+	+	+	+	+	+	+	+	+	+
21) Generalizability	+	+	-	+	+	-	-	+	-	+	+	+	+	-	-	-	-
22) Funding	+	+	-	+	+	-	+	+	-	+	-	+	+	-	-	+	-
Quality	good	good	average	good	good	average	good	average	good	good	good	good	good	good	good	good	average

^a^*NA*, Not Applicable

### Data analysis

In order to analyze the data, following tabulating the extracted data, the process of qualitative synthesis of extracted data was done for systematic review. It should be noted that the ethical considerations in conducting a systematic review including the presentation of scientific materials impartially and by qualified people, avoiding copying and plagiarism and duplicate publications, transparency in the list of authors, and expressing conflict of interest, was observed in conducting this research and the presentation of its results by the research team [29].

## Results

In this systematic review, among 17 final articles which were evaluated, 4 were descriptive studies, 6 were cross-sectional, and 7 were correlational studies. After evaluating the quality of the articles by the STROBE checklist, 13 articles were in the range of good quality [4, 7, 14, 16, 17, 19, 21–27] and 4 articles were in the range of moderate quality [6, 15, 18, 20] (Table 3). No article was given a poor score, so all articles were included in the systematic review.

After reviewing the articles, the needs of single-child couples were divided into four general areas: 1) Financial needs, 2) Cultural needs, 3) Educational needs, and 4) Supportive needs. The support needs included two kinds of social and family support, and the social support included three types of occupational, educational, and medical-health support.

### 1-Financial needs

Financial needs of couples were expressed as government facilities and childbearing incentives in five studies [20, 22, 23, 25, 26]. Organizing and monitoring the increase in prices were mentioned in five studies [7, 16, 20, 21, 24], the perceived needs of couples in the form of occupational needs and eliminating unemployment in four studies [16–18, 22], high child costs in three studies [7, 17, 25], high quality and cheap and accessible child care, prevention of late pregnancy by providing early marriage facilities in three studies [20, 24, 25], housing problem in one study [22] and fertility and healthcare costs and also the cost of genetic abnormality screening in one study [24].

### 2-Cultural needs

Cultural needs including to create the attitude of "child is the capital of life", the culture of need for siblings and the need for friends as the most neglected needs of child, the culturalization of the positive educational effect of peers on the growth and learning and independence of the child, and creating the norm of "multi-child family" were mentioned in 4 studies [7, 18, 20, 23]. Participation in religious meetings (trust and faith in God) was stated in one study [7], the challenge of individualism and social growth and self-priority in one study [6], uncertain and difficult future for children, lifelong responsibility of children for parents, mother's concern for the gender of the second child due to the preference for male gender was also one of the challenges mentioned in couples [7].

### 3-Supportive needs

The supportive needs of single-child couples were in both social and family areas and in line with the harmony between work and family and high quality care of children.

- A- Supportive-social needs which included three areas: 1- Occupational (leave, transfer, flexible working hours), 2- Educational and 3- Medical-health (physical injuries for mother).
  - The supportive needs of the government for accessible, high quality and cheap child care were mentioned in 7 studies that meeting them lead to the highest efficiency in tendency for second child [4, 7, 14, 22, 23, 26, 27].
  - The need to support women's education was mentioned in the study by Rutigliano and Lozano (2022). Prioritizing reconciliation policies between higher education and employment with child care programs is an important factor for desire to childbearing [14].
- B- The need for family support included the participation of spouse, grandmother, and grandfather, which was reported in 3 studies [14, 19, 27]. In these studies, it has been discussed that with the increase in the number of hours men share in housework and child care, the desire to have a second child increases. The need to cultivate men's participation in child care and having egalitarian gender attitudes and the unequal division of child care is the cause of delay in the second birth.

### 4-Educational and counseling needs

Educational and counseling needs included training the consequences of single-child, parenting education, social modeling for men's participation, empowering to improve the quality of marital relationships and attitude towards gender roles in Levin's study (2016) [22], empowering women and men to acquire mutual understanding of the spouse during the infancy in order to prevent the interference of child's responsibility on the quality of marriage, recommendation of health centers to a proper distance between children, the need to use theories such as the theory of planned behavior in creating the correct attitude and desire to childbearing and paying attention to the psychological factors of childbearing [15].

## Discussion

The aim of the current study was to identify the needs of single-child couples, and the findings of 17 studies were reviewed. The current research conclude with the extensive needs of single-child couples regarding childbearing at different levels including four general areas of 1) financial needs, 2) cultural needs, 3) educational needs, and 4) supportive needs, which shows the complexity of single- child couples` childbearing needs.

The important and basic point derived from the studies of single-child behavior is that having single-child is against the desire of people and indeed family and socio-economic restrictions are the basis of this choice. In fact, based on these conditions, people make two decisions; they may delay marriage or birth of their first child in order to overcome problems; also sometimes considering the importance of the culture of marriage and the first child in the society, delay in the second child is considered by families. Also, delay in second child increases the woman's age and fear of the risks and complications of pregnancy at advanced age causes couples to decide to have only one child [30].

In terms of financial needs, one of the important reasons for having only one child is the feeling of economic-social insecurity, although this feeling may not be real and the family is in a good economic and social situation, but the feeling of not being able to control the surrounding environment causes the feeling of insecurity and decides to have only one child. Economic recession causes the choice of single-child. The need to modify socio-economic factors has been shown to play a key role in postponing or forgoing childbearing in France and Italy. Rein Stadler and Fury stated that in the process of deciding to have a second child, when a contradiction appears between mental fertility intentions and objective obstacles in reality, couples experience second child fertility anxiety [31, 32]. Liebenstein used economic theory to construct a cost-benefit model for children. From this point of view, the main cause of second child fertility anxiety is the cost of raising children, that is, if the expected costs of the parents are more than the benefit of the child, the parents may decide to remain as single-child [33].

The need to eliminate unemployment in the desire to single-child has been mentioned in the studies [16, 34, 35]. Kreinfeld (2014) mentioned the need to eliminate male unemployment as a factor in postponing the first and second childbearing in both Denmark and Germany, and noting that fertility during periods of unemployment is lower among women and men with higher education, but not among their less educated counterparts [34].

Economic instability and unemployment on the one hand and the increase in the level of literacy and employment of women on the other hand have increased the single-child trends. Therefore, the need to solve the extra-occupational and intra-occupational challenges of working women regarding childbearing and modifying the childless lifestyle should be raised [36].

In terms of cultural issues, the need to be aware of the level of women's education is considered as the most important factor accelerating the decrease in fertility rate in some countries such as Iran [37]. The need to increase primary education is very important to increase the fertility rate in order to increase women's awareness and improve health services and protect against diseases that cause infertility, but it should be noted that there is a threshold related to income that can be exceeded by increasing the level of education, and "opportunity cost" child birth increases by increase in the income. Although in most studies [14, 37, 38] education increases the age of marriage and delays childbearing and decreases fertility, but a positive correlation between education and fertility was noted in European culture [39]. Regnier stated that it is very important for couples to have a high level of education in Italy, but in France, couples' education is insignificant [35].

The need for favorable media programs and creating appropriate cultural and social models is particularly important for encouraging childbearing [40, 41]. The spread of Islamic culture and virtue of childbearing, modification of values and formation of correct attitudes toward childbearing are very important [30]. Education of religious and religiosity issues reduces individualism and secularism and increases attention to family values [42].

In terms of need for social and family support, an important issue in tendency to have more children is receiving strong social support and kinship network, including spouses, grandmothers and grandfathers who could play an important role in taking care of young children of students and working women. Although the real solution is to establish centers to keep children at the workplaces or near the schools, because in some studies, the non-supportive structure of the society leads to the choice of single-child [43].

In the field of education and the need for psychological counseling, correcting and improving women's attitude and making them willing to have children, counseling people who are hesitant about choosing childbearing, empowering them to coordinate work and family, creating a balance between individual conditions and society, parenting education, avoiding strictness and idealism in parenting are necessary [44, 45]. Moshfegh believes that it is necessary to teach positive value attitudes towards childbearing and the benefits and functions of children in the family and to eliminate the negative values of children during people's education, because the level of education in both groups of working women and housewives has an inverse relationship with the positive values of children and has a positive relationship with children's negative values. The value of a child varies according to the socio-economic developments of each society. In fact, women's desire for social mobility is one of the cultural elements of developed societies, which affects marriage and childbearing behaviors and is reflected by continued education and employment and has an inverse relationship with childbearing [30].

Attitude is one of the most important determinants of reproductive preferences and behaviors, and culture has the most important impact on shaping attitudes. A detailed and scrutinizing look at the complications of childbearing shows the role of culture as a guide for deciding behavioral patterns. Because in every culture, some patterns emerge that remain stable over time and gradually become the cultural norms of the people living in that society [46]. Chavoshi (2016) in a study entitled "Demographic transition and childbearing policies in Iran" stated that according to the educational achievements of the post-revolution generation, their attitudes and aspirations are different from previous generations, and therefore comparing two surveys of changes in women's behavior and attitudes during the last two decades will be very useful regarding gender roles in the field of childbearing [47].

It is noteworthy to compare the needs of single-child couples in different regions of the world. In this regard, it can be said that in European countries, women's financial and socio-occupational support needs and family support for child care were important issues [4, 14]; although Breton stated that in France, education and appropriate culture were reported as needs [18]. In Asia, according to the various studies, financial support and socio-occupational support for women and training the culture of men's participation were suggested as important needs [16, 17, 19, 20, 23, 24, 27]. In Russia, financial needs and provision of a suitable job, women's socio-occupational support, especially formal care for children and training in combining work and family skills were raised as needs; also, improving the quality of the marital relationship was mentioned as a need [22, 26]. In India, teaching parents to avoid excessive parental self-actualization and loss of parental freedom was proposed as an educational needs [6]. While in the United States of America, financial issues were the most important challenge for single-child couples [21].

One of the strengths of the present study was using of a broad search strategy to find relevant studies. According to the present review, this study is the first systematic review on the needs of single-child couples. Recognizing these needs and trying to meet them can modify policies to encourage childbearing and improve the short-term and long-term results of childbearing incentive programs. Identifying these needs, accurate and suitable programs can be designed and implemented in order to help these couples for childbearing. But one of the limitations of this study was that except for English language studies, only articles in Persian were included in the study, which may limit the generalization of the findings. In addition, there was limited data from low- and middle-income countries, which may cause selection bias.

## Conclusion

Considering the challenges and needs of single-child couples, adopting strategies including solving financial needs and unemployment problems, creating a culture of valuing children and men's participation in household affairs, considering women's preferences in order to increase education and employment, training and counseling for childbearing, creating social and family support in line with harmony between work and family and provision of high quality care for children, could be taken into account approaching single-child couples. These strategies should also map long-term plans and can be incorporated in the development of childbearing incentive programs.

### Supplementary Information

**Additional file 1.**

## Data Availability

Datasets are available through the corresponding author.

## Abbreviations

PRISMA: Preferred Reporting Items for Systematic Reviews and Meta-Analyses
MOOSE: Meta-analyses Of Observational Studies in Epidemiology
STROBE: Strengthening the Reporting of Observational studies in Epidemiology
