# Supplementary material for: A systematic review of the childbearing needs of single-child couples
Source: BMC Womens Health. 2024 Feb 1;24:83. doi: 10.1186/s12905-024-02928-0 (PMC10832102; doi:10.1186/s12905-024-02928-0)
Supplement: Supplementary file 1 — Additional file 1. [file 12905_2024_2928_MOESM1_ESM.docx]

Strategy for systematic search of the published literature in PubMed and Cochrane Library database:

PubMed: advanced

Search: **((((only child) OR (one child family)) AND (Motivation)) OR (Reproductive behavior)) AND (Needs assessment)**

((("only child"[MeSH Terms] OR ("only"[All Fields] AND "child"[All Fields]) OR "only child"[All Fields] OR ("one"[All Fields] AND ("child fam"[Journal] OR ("child"[All Fields] AND "family"[All Fields]) OR "child family"[All Fields]))) AND ("motivate"[All Fields] OR "motivated"[All Fields] OR "motivates"[All Fields] OR "motivating"[All Fields] OR "motivation"[MeSH Terms] OR "motivation"[All Fields] OR "motivations"[All Fields] OR "motive"[All Fields] OR "motivational"[All Fields] OR "motivator"[All Fields] OR "motivators"[All Fields] OR "motives"[All Fields])) OR ("reproductive behaviour"[All Fields] OR "reproductive behavior"[MeSH Terms] OR ("reproductive"[All Fields] AND "behavior"[All Fields]) OR "reproductive behavior"[All Fields])) AND ("needs assessment"[MeSH Terms] OR ("needs"[All Fields] AND "assessment"[All Fields]) OR "needs assessment"[All Fields])

Result: 495

Cochrane Library

 Cochrane Reviews matching *** : " childbearing motivation" in All Text**

Result: 31
